# Barriers and facilitators to self-management in people with back-related leg pain: a qualitative secondary analysis

**DOI:** 10.1186/s12998-025-00578-z

**Published:** 2025-05-05

**Authors:** Anna-Marie L. Ziegler, Don Thorpe, Douglas Kennedy, Craig Schulz, Stacie A. Salsbury, Gert Bronfort, Roni Evans

**Affiliations:** 1 Integrative Health and Wellbeing Research Program Earl E. Bakken Center for Spirituality & Healing, University of Minnesota, Mayo Memorial Building C504, 420 Delaware Street, Minneapolis, MN 55414 USA; 2 Palmer Center for Chiropractic Research, Palmer College of Chiropractic, 741 Brady Street, Davenport, IA 52803 USA; 3 Primary Care Services-Whole Health, VA St. Louis Health Care System, St. Louis, MO USA; 4 College of Chiropractic, Logan University, Chesterfield, MO USA

**Keywords:** Self-management, Spinal manipulation, Behavior change, Back pain

## Abstract

**Background:**

Back related leg pain (BRLP) is a problematic subset of low back pain, leading to greater pain, loss of function and health related care costs. While evidence suggests self-management is effective, patient implementation can be sub-optimal. The purpose of this study is to identify barriers and facilitators to self-management for persons experiencing BRLP within the context of a controlled clinical trial and to map these to theory-informed intervention elements that can be addressed by front-line healthcare providers, informing the design and implementation of future theory-driven self-management interventions for this population.

**Methods:**

This study was a qualitative secondary analysis of a 2-site, pragmatic, parallel group, randomized clinical trial (participants enrolled 2007–10) of spinal manipulative therapy (SMT) and home exercise and advice (HEA) compared to HEA alone for persons with subacute or chronic BRLP. We used deductive and inductive content analysis, to describe self-management facilitators and barriers among trial participants, map these to behavior change elements in the Behavior Change Wheel (BCW) Framework, and identify potentially modifiable, theory-intervention elements which may be addressed with guidance by healthcare providers. Baseline characteristics of participants were descriptively analyzed using SAS (University Edition).

**Results:**

Of 40 participants, the majority identified as white (n = 24, 85%) and of non-Hispanic or Latino ethnicity (n = 38, 95%). Average participant age was 57 years old (range 29–80). Frequent facilitators included ease of exercises, knowing how to manage condition, atmosphere created by staff, therapeutic alliance, effectiveness of exercises or treatment, goal of reducing pain, and intentions of continuing exercises. Frequent barriers included time constraints, pain, and lacking confidence in treatment. Barriers were mapped to all 9 Intervention Functions, most common being modelling and education. Frequently identified Behavior Change Techniques included information, feedback, self-monitoring, graded tasks, restructuring, social support, goal setting, reviewing goals, and action planning.

**Conclusion:**

This study identified barriers and facilitators to engaging in self-management for participants in a pragmatic, randomized clinical trial. A rigorous systematic intervention mapping process utilizing the BCW was used for describing what participants need and how their needs may be met. These findings may support the design of future self-management interventions for persons experiencing BRLP.

**Supplementary Information:**

The online version contains supplementary material available at 10.1186/s12998-025-00578-z.

## Introduction

People with chronic low back pain (LBP) experience a myriad of disruptions to multiple facets of their lives, including health, social activity, employment, and identity. LBP is a leading cause of disability [1–4], leading many people to weave together a patchwork of pharmacological, nonpharmacological, and self-management strategies to cope with their pain and symptoms [5, 6]. This is especially true for patients with back-related leg pain (BRLP), a more complicated variation of LBP with symptoms that can be more difficult to self-manage than back pain alone [7–10]. Persons experiencing BRLP pain face greater levels of disability, pain intensity, activity limitations, higher frequency of psychological risk factors, and poorer quality of life than those with uncomplicated LBP [8, 9, 11–13].

Self-management can be defined as an “individual’s ability to manage symptoms, treatment, physical and psychosocial consequences and lifestyle changes inherent in living with a chronic condition” [14]. Clinical practice guidelines (CPGs) consistently recommend patient education, exercise, and self-management as first-line treatments for BRLP due to the impact of these strategies on patient outcomes [15–19]. For example, patients who engage in home exercise programs to manage chronic health conditions may experience a sense of empowerment and reduced dependence on healthcare professionals for treatment [14, 20] Effective self-management however is hard and implementing new self-management strategies on a consistent basis requires behavior change [21, 22], not only on the part of the patient, but also among their clinicians. Healthcare professionals can play a key role in fostering effective BRLP self-management among patients through partnership, support, education, and supervision, and promoting self-efficacy [20, 23–25]. However, health professionals’ practice conventions are often misaligned with guideline recommendations. For example, clinicians prescribe exercise to only half of their patients who might benefit [26] and rarely offer self-care education [27], rather continuing to advocate for rest over active care, and poorly advising patients on the psychological and social dimensions of their experience [26]. Even when health professionals do implement CPG recommendations, patient adherence to exercise and other self-management strategies is sub-optimal [25, 28]. These gaps between ideal and actual treatments necessitate further investigation into how patients and providers understand, recommend, or adopt self-management strategies for BRLP.

Self-management *interventions,* a type of behavioral change intervention, are those that promote the “active involvement of the patient in managing their condition”, and can help the individual learn about and implement health behaviors in their daily lives [29]. However, implementing such interventions can be tenuous [29]. Behavioral change models, for example, Michie and colleagues’ Behavior Change Wheel, highlight sustained behavioral change requires sufficient capability, opportunity, and motivation [30]. The absence of, or barriers within any of these three realms may prevent an individual from engaging in new health behaviors regularly and effectively [21] while facilitating them may support long-term adoption [31–35].

A theory-informed approach to behavior change may allow patients and providers to move from identifying self-management barriers and facilitators to establishing a behavior change diagnosis with its accompanying interventions and expected outcomes [30, 36]. Not all behavior change frameworks are comprehensive, which may lend to heterogeneity among developing and implementing self-management interventions [21, 36]. The Michie and colleagues’ behavior change wheel (BCW) was developed from a systematic review of 19 frameworks of behavior change including nine intervention functions and seven policy categories resulting in a “behavior system” applicable to range of behavior change interventions [21]. Eilayyan et al., and Hurley et al. have undertaken studies to develop LBP self-management interventions utilizing the BCW [31, 34, 37]. However, these studies focused on spinal back pain rather than BRLP, a more complicated and disabling variant of back pain [8, 9, 11]. To further expand the BCW knowledge base, our team undertook a theory-informed evaluation of BRLP self-management interventions. The purpose of our study was to identify barriers and facilitators to self-management for persons experiencing BRLP within the context of a controlled clinical trial and to map these to theory-informed intervention elements that can be addressed by front-line healthcare providers. Our goal was to provide research-based information to improve the design and implementation of future theory-informed self-management interventions for persons experiencing BRLP.

## Methods

This study was a qualitative secondary analysis of a 2-site, pragmatic, parallel group, randomized clinical trial of spinal manipulative therapy (SMT) and home exercise and advice (HEA) compared to HEA alone for persons with subacute or chronic BRLP [38, 39]. Primary outcomes, the trial protocol, and a qualitative analysis examining what patients valued about SMT and HEA are published [38–40]. We used a multi-step analytic approach to describe self-management facilitators and barriers among trial participants, map these to behavior change elements in the BCW Framework, and identify potentially modifiable, theory-intervention elements which may be addressed with guidance by healthcare providers [30, 36]. The clinical trial was approved by the Institutional Review Boards at each research center and registered with clinicaltrials.gov (NCT00494065). Trial participants provided written informed consent. The current study was deemed exempt by the [University of Minnesota Institutional Review Board] (11/08/2021, STUDY00014393). We followed the Standards for Reporting Qualitative Research (SRQR) to prepare this report [41].

### Settings and participants

Participants were enrolled between 2007 and 2010 at the research clinics of Northwestern Health Sciences University (Bloomington, MN, USA) and Palmer College of Chiropractic (Davenport, IA, USA). Participants (N = 192) experiencing chronic BRLP were allocated to receive SMT + HEA (n = 96) or HEA alone (n = 96) for 12 weeks [38, 39]. This secondary analysis included a sample of the participants allocated to SMT + HEA, as this group received the intervention most representative of what chiropractors could deliver in practice.

### Interventions

Trial interventions salient to this analysis included participant receipt of an adapted *Back in Action* book [42], which emphasized the biopsychosocial approach to management of back pain, and 4, 60-min individual sessions of HEA, delivered by chiropractors, exercise therapists and a personal trainer. HEA sessions reviewed information about BRLP causes, prognosis, self-care, and activity encouragement. Participants also received individualized instructions and supervised practice for positioning, stabilization exercises, and spine posture awareness during activities of daily living. Interactions with study clinicians (chiropractors) reinforced this training [38, 39].

### Transcript selection methods

At Week 12, 174 participants completed the self-reported outcome measures, followed by an optional, audio-recorded, semi-structured interview. Interviews were conducted in person by study coordinators and followed an interview schedule with open-ended questions related to satisfaction with care, changes in pain, and feelings toward the home exercise program and chiropractic treatment (Additional file 1) [38, 40]. Of the 174 available, this analysis utilized 40 randomly selected, de-identified, transcripts from participants allocated to SMT + HEA who: (1) completed the study intervention and (2) provided an intelligible response of more than a yes or no answer to at least 1 relevant interview question. Random selection was conducted using a random number generator to minimize selection bias. The lead author (AZ) verified transcripts against these eligibility criteria with the first 40 transcripts meeting criteria used in the analysis. A second researcher (CS) verified eligibility determinations. Transcripts were uploaded to NVivo® v12 (QSR International Pty Ltd, Victoria, Australia) for analysis.

### Secondary data analysis methods

Baseline characteristics were descriptively analyzed using SAS University Edition (SAS Institute, Cary, NC). Three coders (AZ, DT and DK) independently conducted the qualitative analysis using a deductive and inductive content analysis approach [43, 44].

## Coding process

### Codebook development

The team coded for barriers and facilitators to BRLP self-management, using a codebook previously developed for a similar study [30], which was iteratively adapted for this analysis over 3 consensus meetings. The codebook was based on the BCW, Theoretical Domains Framework (TDF) and the Capability, Opportunity, Motivation Behaviour Model (COM-B) and included operational definitions and example statements for each domain (Additional file 2). The COM-B model provides a framework for representing 3 conditions needed for behavior change: (1) Capability is a person’s physical and psychological capacity for engaging in the specified activity; (2) Opportunity represents the social and contextual elements that make making engaging in the behavior possible; and (3) Motivation consists of the beliefs and emotions/impulses directing behavior [21]. The TDF consists of 12 domains synthesized from behavior change theory constructs and support the development of each COM-B element (Additional file 2) [21, 30]. While the COM-B is a useful screening tool, the TDF allows assessing behavior and identifying needs at the individual level, establishing a behavioral diagnosis [30].

### Coding process

Our initial coding process utilized a deductive content analysis approach, coding and mapping directly to the pre-defined codebook. AZ coded all 40 transcripts, with DT coding every other transcript. Both AZ and DT were chiropractors engaged in graduate level research programs. DK, a faculty and research team member experienced in qualitative research and familiar with the TDF and COM-B model, co-coded 5 transcripts with DT before tapering to every 2nd, then every 3rd transcript. Consensus meetings occurred after coding the first 2 transcripts, then after every 10th transcript, for a total of 7 consensus meetings. Consensus meetings included reaching agreement on coding and when needed reviewing clarifying examples ensuring adherence to the codebook. Additional team members (CS and RE) were available to resolve coding discrepancies and confirm accuracy of findings.

After mapping barriers and facilitators to the TDF, it was noted each domain could benefit from further characterization. An additional round of coding was undertaken utilizing an inductive approach for further characterizing the types of barriers and facilitators within each TDF domain. Coding was done within NVivo® v12 (QSR International Pty Ltd, Victoria, Australia) for data organization purposes, no auto-coding was utilized. This also allowed for tracking and quantifying the frequency of each domain and theme. Representative quotes are reported for each theme.

We used the TDF to link BRLP self-management barriers and facilitators with intervention functions (IFs). Intervention functions are broad categories within the behavior change wheel (BCW) by which an intervention can change behavior [30]. There are 9 IFs: (1) education, adding knowledge or understanding; (2) persuasion, facilitating positive or negative feelings or actions; (3) incentivization, using an expectation or reward; (4) coercion, using expectation of punishment or cost; (5) training, imparting skills; (6) restriction, using rules for reducing or increasing opportunity for engaging in the target behavior; (7) environmental restructuring, changing physical or social context; (8) modelling, providing an example; and (9) enablement, facilitating means or removing barriers for increasing capability. The intervention functions link from the COM-B and TDF providing a theory-informed way to move from behavior diagnosis to facilitating change [30]. Once IFs are linked, behavior change techniques (BCTs) can be identified. Behavior change techniques are “an active component of an intervention designed to change behavior” and can be linked to both the TDF and intervention functions [30]. Identifying BCTs occurred by 1 author applying the APEASE criteria while also accounting for most commonly utilized BCTs and applying clinical judgement for types of barriers and facilitators identified [30, 36, 45]. The APEASE criteria considers affordability, practicability, effectiveness and cost-effectiveness, acceptability, side effects/safety and equity of the proposed BCT [30, 46].

## Results

### Baseline characteristics

Baseline characteristics are presented in Table 1, and generally aligned with those reported elsewhere [39, 40]. Most participants identified as white (85%) and of non-Hispanic or Latino ethnicity (95%). On average, participants were 57 years of age (range 29–80) and had an average body mass index (BMI) of 28.1 (SD 5.4). Most participants engaged in light (32.5%) or moderate (50%) physical activity in their daily routines.

**Table 1:** Characteristics of included participants who received spinal manipulative therapy and home exercise and advice program (n = 40)

Age, years, mean (range)	57.1 (29–80)
Body Mass Index (BMI), mean (SD)	28.1 (5.4)
Gender, n (%)	
Female	23 (57.5)
Male	17 (42.5)
Race, n (%)	
American Indian or Alaska Native	3 (7.5)
Asian	1 (2.5)
Black or African American	2 (5.0)
White	34 (85.0)
Ethnicity, n (%)	
Hispanic or Latino	2 (5.0)
Not Hispanic or Latino	38 (95.0)
Marital status, n (%)	
Married or living with significant other	26 (65.0)
Divorced or separated	5 (12.5)
Widowed	6 (15.0)
Never been married	3 (7.5)
Highest level of education, n (%)	
High school graduate or GED	5 (12.5)
Some college or other program, no degree	9 (22.5)
Associate degree (academic or vocational)	9 (22.5)
Bachelor’s degree	8 (20.0)
Master’s degree	6 (15.0)
Professional or doctoral degree	3 (7.5)
Employment status, n (%)	
Currently working full-time	18 (45.0)
Currently working part-time	5 (12.5)
Not currently employed	3 (7.5)
Take care of house or family	3 (7.5)
Retired (not due to health problems)	9 (22.5)
Disabled and/or retired because of back or leg problems	1 (2.5)
Other	1 (2.5)
Household income, n (%)	
Did not answer	4 (10.0)
$10,000–24,999	3 (7.5)
$25,000–34,999	5 (12.5)
$35,000–49,000	7 (17.5)
$50,000–74,000	8 (20.0)
$75,000 or more	13 (32.5)
Amount of physical activity in daily routine, n (%)	
Very light	3 (7.5)
Light	13 (32.5)
Moderate	20 (50.0)
Heavy	3 (7.5)
Very heavy	1 (2.5)
Duration of leg pain, n (%)	
2–4 weeks	1 (2.5)
More than 4 weeks	39 (97.5)
Typical severity of leg pain in past week, mean (SD)	5.6 (1.7)
Typical severity of low back pain in past week, mean (SD)	5.5 (2.6)
RMDQ*, mean (std)	10.9 (4.1)

*Roland Morris Disability Questionnaire, scored 0–24 with higher scores indicating more disability

### Capability

Table 2 displays domains, operational definitions, and corresponding themes relating to physical and psychological capability. Physical capability refers to the participant’s physical ability or skill to perform self-management activities for BRLP. Physical ability or skill was mentioned by 19 participants (48%). A singular barrier to self-management, difficulty performing exercises, was mentioned by 4 participants (24%). Exercises feeling simple to perform was mentioned by 13 participants (76%) as facilitating self-management.

**Table 2:** Barriers and facilitators relating to physical and psychological capability

Theoretical domains framework	Overall cases (N)	Facilitators (N)	Barriers (N)	Themes Facilitators and Barriers (N)
Physical capability				
*Physical Skills* Individual mentions ability and proficiency for a physical skill acquired through practice impacting self-managing (symptoms, treatments, etc.) [an ability of proficiency acquired through practice]	19	17	4	*Facilitators* Exercises were simple (13) “I liked that it was fairly simple to do…they weren’t real complicated” (10,272) Tailored to patient’s abilities (3) “She, compensated my exercises so I didn’t have to use my neck as much or I supported my neck so that I didn’t get any pain.” (10,343) Gaining new skills for future application (2) “[Name of Dr.] explained to me about preventing it, preventative maintenance for my lower back about…for example, when I do bend now, instead of just bending down, what I do is the golfer’s kick.” (14,953) *Barriers* Difficulty performing exercises (4) “I mean I couldn’t do the exercises” (13,246)
Psychological capability				
*Knowledge* individual mentions an awareness of the existence of something impacting self-managing (symptoms, treatments, etc.) [an awareness of the existence of something]	24	24	4	*Facilitators* How to manage condition (11) “Too much bending or whatever. I mean, you know, bending over. So, but I’ve learned thr-through conversations with chiropractor and, and the physical therapist, I suppose it would be that gave me the exercises. I’ve learned how to do some things differently. So, you avoid some of those occurrences, so you didn’t have pain. So, and of course because of my age, I going ta, it’s not going to go a hundred percent, because of my age and I know that. But it certainly has helped me.” (13,097) Understanding exercises (9) “Things that I can do to strengthen my um, I haven’t had any exercise programs that I liked, what exercise I should be doing to strengthen my back um, and I liked all the handouts they give me, I’m a visual person so I liked to have it with the reference. Once I’m done I have something to go back to that I can use. I think that was probably the manual or the three ring binder with the exercise I think was the biggest thing for me.” (15,047) Being provided knowledge, general (8) “It’s more than I expected. I’m just amazed at how a little bit of good knowledge can make you take care of yourself and, and feel better. Cause’ I had completely stopped exercising.” (10,151) Understanding condition (4) “Mmmh. Yes, I guess so, cause’ it did ultimately eliminate…do I have sciatica? No! And I can move on and hopefully… get treatments somewhere else for what, what is the problem.” (13,631) Managing expectations (2) “There were a couple occasions when I was kind of sore afterwards…I think it’s kind of like opening a hornet’s nest, sometimes you work with muscles that maybe aren’t used to being worked with and so you know it, there are, there are a few occasions when I was pretty pain- you know, I was pretty painful afterwards, but that does goes away.” (13,751) *Barriers* Not knowing how to manage condition (3) “It was mostly the legs, that were giving me the most problems. I…you kind of know what to do for your back… if something annoys your back, you stop doing it. You know, you do things differently. But your legs, what do you do with your legs you know?…You got to use them.” (13,097)0 Not understanding exercises (1) “The reason for that is because, you know, although I have the picture there, I don't, it’s not necessarily, it’s not the same as a video or somebody standing there and correcting you, “Don’t go this far up, uh, don’t do this.” I was doing them wrong the first week or so” (10,140)
*Cognitive and interpersonal skills* individual mentions an ability or proficiency in a cognitive skill acquired through practice impacting self-managing (symptoms, treatments, etc.) [an ability of proficiency acquired through practice]	6	5	2	*Facilitators* Recognizing and communicating a need (3) “…and they’re, easy to follow and I think one thing that I would have liked and I kind of did on my own and stuff, I talked to [name of Dr.] about it but I started using like hand weights and stuff…going along with the exercises and stuff which I think helped me a little bit more…” (13,657) Recognizing and reorganizing schedule for fitting in exercises (1) “I think it just made me, again I’m an organized person and it got me to be a little bit more organized because I knew what I had to do at a certain amount of, you know. Even though I got home late at night or whatever the case may be, I still had to get it done.” (10,014) Recognizing correct performance of exercises (1) “Well, then I noticed I had been doing them wrong, because when I did it the way he told me to I didn’t feel the level of discomfort pain that I had been feeling.” (10,140) *Barriers* Not recognizing correct performance of exercises (1) “…I guess I never knew quite if I was engaging the right muscles (cleared throat).” Interviewer: “Ok. When you were doing…” Patient: “By myself.” (13,576) Difficulty pacing exercises (1) “Well, I think at first I was over-exerting myself. And then I talked to the doctor and he took me off a couple of em’, and said, “Don’t over-exert” because I just thought they weren’t strenuous enough. So I had a misconception on, on the exercises and I think I did hurt myself the first couple weeks…” (10,014)
*Memory, attention, and decision processes* Individual mentions retaining information, gaining or maintaining focus, or making decisions pertaining to self-managing (symptoms, treatments, etc.) [the ability to retain information, focus selectively on aspects of the environment and choose between 2 or more alternatives]	6	4	2	*Facilitators* Awareness of when to incorporate self-management strategies (2) “I, um, was given instructions how to lift things and stoop and bend and, um…You know I keep them in mind now when I do those activities” (13,424) Being disciplined (1) Interviewer 4a. “And what did you like best about the home exercise program you had in the study?” Patient: “The discipline of me just doing them. I said I, I think I’ve missed one day of exercise since I started. I mean one, one portion of the day” (10,151 Incorporating into routine (1) “That I was introduced to a few new exercises that seemed to help. That I was able to combine the recommended exercises with other exercises that I’d already been doing on a daily basis along with yoga and stretching…” (10,178) *Barriers* Difficulty deciding to exercise (2) “I’m not an exerciser so, I mean, that would, for me, it’s just the idea of exercising. It’s having to discipline yourself to do this on a regular basis. They weren’t strenuous, there were not, I mean so much that it was overwhelming. But it’s just the idea, I mean like for me, any type of exercise I just don’t like to do it.” (13,537)
*Behavioral regulation* Individual mentions something pertaining to managing or changing self-managing behaviors [anything aimed at managing or changing objectively observed or measured actions]	4	2	2	*Facilitators* Changing activity to reduce pain (2) “The…that how much it’s changed it’s just affected my life because, before I would just kind of like sit and wonder what was happening to me, I was getting older. Now, if it’s hurting I get up and do some stretches and I look forward to doing my exercises three times a day” (10,151) *Barriers* Choosing other activities over assigned exercises (2) “There near the end about week nine or so I started lacking, slacking off because I felt that my swim routine and my calisthenics and, what I was doing as far as pushups and chin ups and that kind of thing was contributing to about the same kind of exercise that was called for by the regime to get em’ in.” (10,307)

Psychological capability refers to a participant’s knowledge, cognitive and interpersonal skills, and memory, attention, and decision processes in self-management behaviors for BRLP. Twenty-four participants mentioned a knowledge barrier, such as not knowing how to manage their condition (13%), or at least 1 facilitator, including knowing how to manage their condition (49%), understanding their exercises (38%), and general knowledge gained during the study (33%). A barrier or facilitator pertaining to cognitive and interpersonal skills was mentioned by 6 participants (15%). Half of these participants cited recognizing and communicating a need as an enabling factor (n = 3; 50%). Barriers included not recognizing correctly performing exercises (n = 1; 17%) and having difficulty pacing exercises (n = 1; 17%). Six participants cited a barrier or facilitator relating to memory, attention, and decision processes. Two participants (33%) cited being aware of when to incorporate self-management strategies as an enabling factor. Difficulty making the decision to exercise was mentioned by two participants (n = 2; 33%).

### Opportunity

Table 3 displays domains, operational definitions, and corresponding themes relating to environmental context and resources, as well as social influences to enact self-management behaviors for BRLP. A facilitator or barrier related to environmental context and resources was cited by 38 participants (95%). Majority of participants cited the atmosphere created by staff as a facilitator for seeking treatment (n = 21; 53%). Others described the exercise schedule (n = 10; 26%) such as the progressive increase in difficulty, and portability of exercises allowing their use anywhere (n = 7; 25%). Lesser mentioned were affordability of treatment (n = 5; 13%), finding handouts helpful (n = 5; 13%), and flexibility of scheduling (n = 5; 13%). Environmental barriers were mentioned by 19 participants. Twelve participants cited time constraints as a barrier, often described as impacting performing exercises (32%). The driving distance to appointments (n = 3; 8%), the exercise schedule in terms of number of prescribed exercises (n = 2; 5%), lacking video or in-person visual demonstration (n = 2; 5%), and having space constraints for performing exercises (n = 2; 5%) were additional barriers.

**Table 3:** Barriers and facilitators relating to physical and social opportunity

TDF domain	Overall cases (N)	Facilitators (N)	Barriers (N)	Themes Facilitators & Barriers (N)
Physical				
*Environmental context and resources* Individual mentions something in their personal situation or environment discouraging or encouraging the development of skills, abilities, independence, social competence, and adaptive behavior for self-managing (symptoms, treatments, etc.) [any circumstance of a person's situation or environment that discourages or encourages the development of skills and abilities, independence, social competence, and adaptive behavior]	38	34	19	Atmosphere created by staff (21) “I really appreciated the fact that not only was everyone professional and really kind but that they was incredibly prompt and, punctual and proficient and, there handling of me from all the way through to everybody, it was, they were great. The staff was fantastic.” (13,709)
				Exercise schedule (10) “I like the fact that it increased slowly over time. We didn’t start out with a real intense program, we started out less” (10,272)
				Portability of exercises (7) “That I could do ‘em at home. (Laughs) Pretty much do ‘em, pretty much anywhere.” (11,170)
				Affordable (5) “Oh, I just really that simple that’s gonna cost me nothing.” (11,170)
				Handouts were helpful (5) “That I was able to do it on, on my own time. And that the pictures were given to me, cause’ again I’m visual and so to me it’s, it’s easier to look at, look at the pictures and be able to do them.” (10,140)
				Appointment scheduling flexibility (5) “How, convenient or how adjustable, when I needed to make changes in the time I could arrive or if I needed to change an appointment, how convenient those were” (10,272)
				Visit frequency (3) “The frequency of the visits and chiropractic adjustments” (10,178) Having time (3) “The fact that I am retired I do have the time” (10,307)
				Equipment (3) “It might sound silly but just the, there was a, a lotion that was used which was great but, just the smell of it…” Center accepts prior imaging (1) “The fact that you accepted the, the x-rays that I already had done.” (10,307)
				Changing providers (1) “What did I like least about that one? I guess changing, at the time when I changed doctor’s right in the middle. It was, kind of gone through all the explanations, we’d been doing everything up to a certain point and then we made a full change when we went from one doctor to the other. In the end, it probably ended being the best thing that could happen because I ended up getting’- some results, but at the time it was a little frustrating.” (10,272)
				Time constraints, barrier (12) “The whole exercise part though it, I know they talked about wanting to do it 3 times day…But with my schedule they said two was ok…I can’t imagine too many people would do it more than twice a day…”(10,289)
				Driving distance, barrier (3) “I think probably for, its, I have to drive here so far and so by the time I got here I was so stiff. So I think it wasn’t really the actual adjustments it was just that getting here that was probably the biggest hassle because I live northwest from here so it was a little bit of a drive.” (15,047)
				Exercise scheduled, barrier (2) “The number of exercises and the amount of time.” (laughs) “I could say that I think if I-being a programmer, I would have started heavy then worked more toward light-then carrying that same volume throughout-throughout the whole…It, it got to be at the end, it got you know-just to be, repetition but yet, uh, monotonous” (10,509)
				Lacking video or in-person visual demonstration, barrier (2) “I guess it would have to be my same answer from before. The reason for that is because, you know, although I have the picture there, I don't, it’s not necessarily, it’s not the same as a video or somebody standing there and correcting you,” (10,140)
				Space Constraints, barrier (2) “to do the things but when I’m…have my grandson with special needs then, of course that, you can’t lay on the floor but then he thinks you’re playing a game and he’s going to get on top of you to wrestle or something.” (13,097) Working a desk job, barrier (1) “I mean the change in the pain the-the…the reduction in the pain. And that, and that of course goes up and down because sitting is really bad for me and I sit the majority of time at work.” (14,479)
				Length of treatments visit wasn’t long enough, barrier (1) “The time wasn’t long enough.” (13,537) Visit Frequency, barrier (1) “I didn’t get called enough I’d once that my appointment had been cancelled. So I showed up, that’s about it.” (13,576)
Social				
*Social influences* Individual mentions interpersonal interaction or influence causing their thoughts, feelings, or behaviors towards self-managing (symptoms, treatment, etc.) to change. [those interpersonal processes that can cause individuals to change their thoughts, feelings, or behaviors]	24	24	2	Therapeutic alliance (22) (caring, competency, professional, adaptability, honesty) “I think again, there it’s the, I guess the feeling that, he really- he did care and he was going beyond what really what might be expected. And, that is something that I think is fantastic. That, to me, that you know, demonstrates that the person um, is sincerely interested in not only, their field of medicine or what they’re doing but in helping the patients you know, work through whatever issues they have” (13,537)
				Practitioners providing knowledge (3) “to pick one thing in particular, each doctor that I saw provided an excellent, excellent explanation as to what they were doing with me, why they were doing it, and the fact that somehow it validated the pain that was in my head.” (10,140)
				Practitioner giving recommendations (2) “Person I worked with. She's was very comforting, very helpful. Went above and beyond, my appointment. She, if I'd see her when I was here, she would make recommendations and we’d discuss, new options.” (13,767)
				Changed views towards conservative care, practitioners (1) “I told one of the doctors that I had always heard that chiropractors were crack pots… and now I know better!” (10,151) Family Opinions, barrier (1) “My husband, my people think I am nuts, my family thinks I am nuts because I would just off, absolutely, leave and go do ‘em (patient laughs)” (10,151) Therapeutic alliance, barrier (1) “And then, even after just a massage and then after that she said is, you know, its ridiculous you’re even coming in. Making the drive” (13,246)

Twenty-four participants (60%) mentioned a barrier or facilitator related to social influences, with each of the 24 (100%) citing a facilitator and 2 of the 24 participants (8%) citing a barrier. All facilitators were related to the influences of practitioners in the trial. The therapeutic alliance was cited as a facilitator by 22 of the 24 participants (92%) and was often described as practitioners as being caring, competent, professional, honest, and able to adapt. Barriers were less frequently mentioned in this domain with 1 participant (n = 1; 4%) citing family opinions, and 1 participant a break in the therapeutic alliance (n = 1; 4%).

### Motivation

Table 4 displays domains, operational definitions, and themes related to automatic and reflective motivation. Reinforcement was cited by 21 participants (53%) as either a facilitator or barrier. The effectiveness of exercises encouraging continuation of utilizing exercises was described by 12 participants (57%). Similarly, effectiveness of treatments was cited by 4 participants (19%) as a facilitator. Pain was described as both a facilitator and barrier to self-management. As facilitator, pain was described as prompting engaging in self-management strategies by 2 participants (10%). Pain posed a barrier for 5 participants (24%) with pain upon exercising discouraging engaging.

**Table 4:** Barriers and facilitators related to automatic and reflective motivation

TDF domain	Overall cases (N)	Facilitators (N)	Barriers (N)	Themes Facilitators & Barriers (N)
Automatic				
*Reinforcement* Individual mentions something increasing the probability of self-managing behaviors (symptoms, treatment, etc.) via a dependent relationship, or contingency, or responding to a stimulus. [increasing the probability of a response by arranging a dependent relationship, or contingency, between the response and a given stimulus]	21	19	6	Effectiveness of exercises (12) “Sometimes I resent having to do it twice a day (chuckles), but I still do because it makes me feel better.” (14,479)
				Effectiveness of treatment (4) “Well, pointing out to me again um, how to minimize or treatment the knots in my muscles, and as I said even though shortly after the chiropractic treatments that I would get more pain. It would go away and I think overall that there was a benefit there. So even though that, you know twenty-four hours would be a bit of a price to pay I guess after that I always felt like that it was, I was stronger and-and better able to do more with that, with less pain later…So I think the mobility of it again so, you know, I think it was comparable to, what I had described as an improvement overall that I had I think that…was probably from that.” (13,709)
				Organization or schedule (2) “I think it just made me, again I’m an organized person and it got me to be a little bit more organized because I knew what I had to do at a certain amount of, you know. Even though I got home late at night or whatever the case may be, I still had to get it done.” (10,014)
				Pain (2) "Always, I take medication I you know, always, when I wake up I feel pretty rotten. You know, when I'm rotten, pretty pain, you know, I feel a lot of pain" (13,246) “Well on an on going basis I had noted during the course of the treatment that, that the afternoon and the day following chiropractic treatment my back would be more sore, but of course the question was always do you have to get a little bit more pain short term to find long term improvement. And, the same thing was true with the exercises if I did a lot but again my back pain has always been, related to physical activity. On the other hand if you don’t do any physical activity then the stiffness and pain spreads to other parts of the back. So, so you know it was, but that was usually the day or so after. Over the course of the treatment longer term I felt there was, you know, a significant improvement probably about a twenty-five percent improvement. I judge that not by the amount of pain but by the amount of activity that I can do. For the same amount of pain (chuckles).” (13,709)
				Maintaining working (1) “I feel that I did get something out of this and, I’m gonna use what you guys gave me for the rest of my life, to feel better. And, so I can keep working. Cause that was the main thing is that, I wanted to be able to continue to work. And I think I can do that now” (14,593)
				Effectiveness of ergonomic changes (1) “The adjustment, the heat…so many things that [name of Dr.] explained to me about preventing it, preventative maintenance for my lower back about, you know, for example, when I do bend now, instead of just bending down, what I do is the golfer’s kick. Which makes it a lot better, to bend down. I mean I can do that all day. You know versus what I was doin prior to that was like at work we have a sanitizing bucket, that sets on the floor that we clean the tables with. And I used to take that bucket and set it up on the table to keep from bending down, but now that I do the golfer’s kick. Oh its no problem. I bend down and get it, and that really feels good on my lower back.” (14,953)
				Confidence (1) “It’s been wonderful. Overall for my attitude about my back problem. With giving me skills to be able to go forward. Just overall, I’ve it’s made my life one hundred percent better. I’m very grateful for the study…I feel much more confident in taking on exercise by myself. And building my core muscles and knowing that once I build those muscles it’s going to relieve the back pain. It won’t go away but at least I’ll be able to do my normal activities everyday.” (13,767) Wanting to progress (1) “Knowing that I would, feel more muscle problems, going through those, each of those steps. But I knew that it was going to be a positive turn around on the other side so I just kept going. It’s been a roller coaster, you know it heightened as it goes and as you go, but of course it’s been you know knowing that I can get to the next level has pushed me through that.” (13,767)
				Pain, barrier (5) “Well, because it irritated, you know it hurt, and that I got scared to do em” (13,246) Psychological impact of capabilities, barrier (2) “Well I guess what I liked best about it is just trying to have a discipline of, of sticking to something. There was one exercise that was always difficult for me, I know they said well don’t worry so much about that one, so that was good. You know if its something you just really detest doing then you’re not gonna do it” (14,864)
				Other medical conditions, barrier (1) “And then, if there’s some flakes in there it’ll make me dizzy and that made the exercise much harder for about three weeks, and that thing to I think is what caused me not to come here last week to do this cause member I said I didn’t feel I felt dizzy that day. And at the middle of the day in the, I still, I saved that little, that, it was a flake that came out of my ear by the end of that day, and I know that’s what had done it. And if I had, I felt if I had come in here that day to do it I just probably wouldn’t have been able to do everything. Cause my, my…balance level was not perfect…But I did have that right before Christmas and it was around my last HEP appointment I had that. So, so that definitely did interfere. And it might be too, that my, eh, my, o- overall stuff would have been, would be a little bit better if that hadn’t been in there.” (14,864)
				Change in environment, barrier (1) “I mean I live alone, so I have, you know, I have, when I’m home and not busy I have plenty of time to, you know to do the things but when I’m…have my grandson with special needs then, of course that, you can’t lay on the floor but then he thinks you’re playing a game and he's going to get on top of you to wrestle or something...” (13,097)
*Emotion* Individual mentions a reaction pattern involving a experiential, behavioral, or physiological element stemming from or impacting self-managing (symptoms, treatment, etc.) [a complex reaction pattern, involving experiential, behavioral, and physiological elements, by which the individual attempts to deal with a personally significant matter or event]	3	1	2	Validation (1) “I don’t know that I know to pick one thing in particular, each doctor that I saw provided an excellent, excellent explanation as to what they were doing with me, and why they were doing it and the fact that somehow it validated the pain that was in my head.” (10,140) Guilt, shame, barrier (2) “Just that, that feeling of guilt… of not finding, making the time to, to do it.” (13,631) Fear, barrier (1) “Well, because it irritated, you know it hurt, and that I got scared to do em” (13,246)
Reflective				
*Goals* individual mentions a mental representation of outcomes or end states they want to achieve for self-managing (symptoms, treatment, etc.) [mental representations of outcomes or end states that an individual wants to achieve]	37	36	6	Reduce pain (29) I thought that it would, that the pain would dimin…diminished. (10,140) General improvement (8) “I took into account, my interactions with staff. I took into account, the results that I felt that I achieved during the program and the progress made, and whether it I guess whether it achieved a level of improvement that I felt was significant or satisfactory.” (13,709)
				Move easier (7) “I didn’t know what to expect. I did have some inclination that, getting more mobility and strengthening my back and abs and core and, would help me overall. Yes.” (10,307)
				Improve strength (4) “Well, to be honest with permanent nerve damage, with a fourth herniated disc I didn’t expect that you would be able to improve the pain a lot and wouldn’t be able to improve the tingling and loss of feeling in my leg at all. But I did expect that there would be some strengthening of the back which did occur.” (10,178) Improve sleep (3) “I think my biggest thing I took into consideration was my improvement in sleep.” (14,479)
				Increase ability to exercise (3) “Well I think I feel a lot better. I am doing more than I was doing before. I, you know the pain has been dramatically reduced. I am able to, you know, walk three miles where as…three months ago I wouldn’t have even considered doing that. So, I am very pleased with how, I’ve changed and I think a, Dr. Taylor mentioned it, he says your like a model patient here. Because I did put out, I, you know, knocked weight off I, uh, did the heavier exercising, I did all the things your going to do and as a result I got the desired results. That all. You get out what you put into it. Not everybody, not everybody does that because of time. I am retired now, so I can, I can do it. And be focused.” (14,428)
				Improve health (2) “One of the key factors, is, the feeling that I did get, I was getting better. You know there were, there was some positive health related things happening” (13,537) Increase activity level (2) “…and I can do twenty-five percent increased activity, involving the lower back, for you know with less much less pain then I had before. So about a twenty-five percent improvement that’s again how I describe it I know I’m going to pay a price…if I do very much but how much can I do and for what cost and that’s definitely better now.” (13,709)
				Lose weight (2) “I think it is actually a little bit more than what I had expected. I am going to continue the weight loss, hoping to lose another forty pounds. Plan to keep the back exercises up…just because it strengthens, and I now know what I can do and what I can’t do. Keep the exercising up.” (14,428)
				Improve muscular restrictions/contractures (1) “On my leg, even though my leg does ache from time to time, you know before when I would like feel the back of my leg. I could feel like, um…bump, lumps kind of in the muscle. And that, I have none of that anymore. So that was a big improvement. And that’s probably the biggest thing I can mention.” (14,864)
				Less stiffness (1) “It cost me nothing, and it, they, when I first came I couldn’t hardly sit down or walk for that matter and now I’m back to work pretty much almost back to normal…Well, I wake up in the mornings I’m a little stiff but other than that, I do the same things I normally do.” Interviewer: “So less stiffness?” Patient: “Yeah.” (11,170)
				Maintain current level/don’t get worse (1) “Well, because like I said, I think it helped strengthen my back. It gave me some new exercises to use in helping with my own physical therapy and care at home to try to keep the back from getting any worse.” (10,178) No change, barrier (6) “…going in I really didn’t expect any change and that’s about where I ended up” (10,272)
*Optimism* Individual mentions confidence in outcome, attaining goals, or physical or mental capabilities for self-managing (symptoms, treatment, etc.) [the confidence that things will happen for the best or that desired goals will be attained]	33	17	9	Chiropractic treatments work (13) “I didn’t have any expectations. I was hoping that, that it would help, and it certainly did.” (13,091) Exercises work (7) “Mm…eh, I think that you know, learning the exercises and I can once I’m able to do em I think that they’ll help.” (13,246) Can self-manage (3) “I feel so much better. It’s just, it’s given me the confidence that I can help myself by listening to what they tell me to do” (10,151) Provider recommendations work (1) “Every, everyone of the, I’m going to call them practitioners, I don’t know what else to call them had been really great in listening to me and explaining to me what they were doing and, and the remarkable thing is that everything they tell me works.” (10,151) Chiropractic treatments won’t help, barrier (9) “I, I didn’t say you know, actually I usually, I feel worse. You know. Until finally I said, this one doesn’t work. You should try a different one. So then she tried a different one, then finally I, I realized this isn’t gonna help me” (13,246)
*Beliefs about consequences* Individual mentions accepting truth, reality, or validity about outcomes of a physical or cognitive behavior in pertaining to self-managing (symptoms, treatment, etc.) [acceptance of the truth, reality, or validity about outcomes of a behavior in a given situation]	29	24	10	Exercises will reduce pain (11) “Um, it’s been wonderful. Um, overall for my attitude about my back problem. Um with giving me skills to be able to go forward. Um, just overall, I’ve it’s made my life one hundred percent better. I’m very grateful for the study…I feel much more confident in taking on exercise by myself. And building my core, muscles and knowing that once I build those muscles it’s going to relieve the back pain. It won’t go away but at least I’ll be able to do my normal activities everyday.” (13,767)
				Exercising will result in general progress (9) “Well I liked best that since it was at home I could do it on my own schedule and I could do exercises for a short period of time and then take, a break, and do some more later, so I guess those were the main things I…it kept me uh…, I think it did improve my health a little bit because exercise is always good for you and I would tend to feel a little better after I had exercise.” (13,860)
				Chiropractic treatment will result in general progress (4) “They were bene…I think they were beneficial in helping the improvement.” (10,289) Chiropractic treatment reduces pain (2) “I’m not really getting any leg pain now or very little. That’s how I answered it at least, so. My low back is still stiff, but not like it was and…It’s mainly when, the chiropractor was doing the flexion and that kind of thing, so.” (10,289)
				Weight loss will improve pain (1) “I don’t know that I had any great expectations when I came in. I guess I was rather skeptical, but I, I do think there is some improvement, as we’ve discussed, you know, some of it is undoubtedly due to the fact that I’ve lost weight over that same period of time. But I do feel that the exercises and so on have helped.” (10,245)
				Because of type of condition nothing will help the pain, barrier (3) “Well, I figured with the type of pain I had, a sciatic nerve type of thing, yeah, I didn’t think there was much you could do with that. You know, it’s just something that, that came about and you, you cope with it. But I found out through the exercises and things that there were things I could, I could by strengthen up the core and try and stand a little straighter and it, it has helped.” (10,343)
				Not having chiropractic care would result in less or no improvement, barrier (3) “Well, I think what it did…I felt that if I didn’t get that side of it, if your back was misaligned all the other work that you do, (laughs), wouldn’t give you the total, the total package I felt being on the chiropractic side in addition to that. I remember [name of Dr.] first comment when he got on me the first day, when he tried to do his first adjustment he bounced back off the wall I was so tight and so by now it, you know you he actually, as I continued on through it he said, ah it just automatically falls into place where were getting there, so… It was a tremendous change from that side of it now if I had still been as tight as a drum all these other exercises wouldn’t have given me as much benefit as I had.” (14,428)
				Exercise needs to be difficult to be effective, barrier (1) “Uh…, well, I think at first I was over-exerting myself. And then I talked to the doctor and he took me off a couple of em’, and said, “Don’t over-exert” because I just thought they weren’t strenuous enough. So I had a misconception on, on the exercises and I think I did hurt myself the first couple weeks…and then he took me off that a little bit and then I, I really, what I do is quite minimal to tell you the truth I think now, but apparently it’s really helped.” (10,014)
				Focused chiropractic treatment has less benefits, barrier (1) “One of the things that I thought about the chiropractic is that, I’m not sure…it’s helpful to…to just spot treat like that, you know sort of like I think, you know we were treating the low back but, you know my pain…radiated all the way up the back and all, [name] really worked on that…the muscle there. I, I just really felt like if they would’ve done chiropractic, you know, treatment…in my mid back. You know, sometimes I think it is all connected. And when you just do one thing like that, then I’m not sure that you get the full benefit…that, that would be my…downside on the chiropractic part.” (13,629)
				Non-prescribed exercises have the same benefit, barrier (1) “There near the end about week nine or so I started lacking, slacking off because I felt that my swim routine and my calisthenics and, what I was doing as far as pushups and chin ups and that kind of thing was contributing to about the same kind of um exercise that was called for by the regime to get em’ in.” (10,307)
				Chiropractic treatment increased my pain, barrier (1) “Okay, oh gosh…you know like I say, that the exercises I was shown, this and other things.. strengthening my core will make it easier, you know that, all made sense and I think it’ll be good. But because my back is worse, I mean I couldn’t do the exercises and, and so the one part of it, is the questionnaire, which I…I don’t know what the answer would be, is uh, none of the chiropractic treatments helped me. Cause basically, you know it took me a while to realize that, well I felt worse after the treatments, you know, usually by that night or the next morning, I kept hoping that the next treatment would make me better, and then when we decided its, I probably have a herniated disc, then we discontinued em, so, if that hadn’t happened, you know they might have helped, my left leg, but once my right leg started hurting so bad… made me realize I didn’t have it so bad. In the beginning. You know so, I, I don’t want it to reflect on the chiropractor or the treatments she gave me, I just don’t think, at this time it was irritating my back instead of helping it.” (13,246)
*Intentions* Individual mentions a conscious decision for performing a behavior, resolving to think or act in a certain way related to self-managing (symptoms, treatment, etc.) [a conscious decision to perform a behavior or a resolve to act in a certain way]	16	16	0	Continue exercising (13) “Well, I just, I think what I liked best is that maybe I’ll, I hope, the fact that I’ll continue.” (laughs) “Regardless of whether it helped my back or my leg…it’s a, it’s a good thing to be doing” (13,081) Being disciplined (3) “It’s having to discipline yourself to do this on a regular basis.” (13,537)
				Resume exercising (1) And then, I’d just start, I’d you know I have stopped doing the exercises. But, I plan to resume them, You know, as soon as I can do so without making my pain worse.” (13,246) Seek additional treatment (1) “Yes, I guess so, cause’ it did ultimately eliminate…do I have sciatica? No! And I can move on and hopefully… get treatments somewhere else for what, what is the problem.” (13,631) Continue losing weight (1) “I think it is actually a little bit more than what I had expected. I am going to continue the weight loss, hoping to lose another forty pounds. Plan to keep the back exercises up. Just because it strengthens, I now know what I can do and what I can’t do. Keep the exercising up.” (14,428)
*Beliefs about capabilities* Individual mentions accepting truth, reality, or validity about their ability, talent, or facility for self-managing (symptoms, treatment, etc.) [acceptance of the truth, reality, or validity about an ability, talent, or facility that a person can put to constructive use]	7	5	2	Able to exercise (2) “It’s been wonderful. Overall for my attitude about my back problem. With giving me skills to be able to go forward. Just overall, I’ve it’s made my life one hundred percent better. I’m very grateful for the study… I feel much more confident in taking on exercise by myself. And building my core muscles and knowing that once I build those muscles it’s going to relieve the back pain. It won’t go away but at least I’ll be able to do my normal activities everyday.” (13,767) Increased activity (2) “Because I was able to, I’m able to do more thing and I learn how to, do more things than I did, from the exercises and the, how I was told how to bend and lift and the strength of how to, you know, the strength that come there so…” (14,105)
				Helping oneself (1) “I feel so much better. It’s just, it’s given me the confidence that I can help myself by listening to what they tell me to do” (10,151) Not capable of exercise, barrier (1) “No I can’t. Can’t even walk a long distance, you know?…I’m good at tolerating pain, but the pain I get running or walking is, you know, way different than just you know, bending.” (13,246) Not capable of being strong, barrier (1) “I would say that I accept that, you know, that I am getting older and I no longer have the strength and endurance and I have other little problems such as the heart problem. I’m doing pretty good. I feel very strongly that I’m in pretty shape, physically.” (10,307)

Goals as a facilitator or barrier was discussed by 37 participants (93%). Thirty-six participants (97%) cited a goal as a facilitator. Goals included reducing pain (n = 29; 78%), general improvement (n = 8; 22%), moving easier (n = 7; 19%), improving strength (n = 4; 11%), improving sleep (n = 3; 8%), increase ability for exercising (n = 3; 8%), improving health (n = 2; 5%), increasing activity levels (n = 2;5%), losing weight (n = 2; 5%), improving muscular restrictions/contractures (n = 1; 3%), less stiffness (n = 1; 3%), and maintaining current level of BRLP (n = 1; 3%). Barriers in this domain included not setting goals or expectations and were mentioned by 6 participants (16%).

Optimism was cited as a barrier or facilitator by 33 participants (84%). Having confidence in the efficacy of chiropractic treatments was discussed by 13 participants (40%), while 9 participants (27%) cited lacking confidence in the chiropractic treatments helping as a barrier. Confidence in efficacy of exercises were discussed by 7 participants (21%). Three participants (9%) discussed their own confidence in ability to self-manage as a facilitator.

Twenty-nine participants (73%) discussed a barrier or facilitator related to beliefs about consequences, with 24 participants (83%) citing a facilitator and 10 a barrier (34%). Beliefs about consequences enabling self-management included believing exercises will reduce pain (n = 11; 38%), exercising will result in general health progress (n = 9; 31%), and chiropractic treatment will result in general health progress (n = 4; 14%). Barriers included participants believing, because of their type of condition, that nothing will help their pain (n = 3; 10%). Others believed not having chiropractic care would have led to less or no improvement in their condition (n = 3; 10%), or that chiropractic treatment focused on the low back was less beneficial (n = 1; 3%).

Intentions for self-management was mentioned by 16 participants (40%). No participants cited a barrier in this area. Intentions included continuing exercising (n = 13; 81%), being disciplined in completing exercises (n = 3; 19%), resuming exercising (n = 1; 6%), seeking additional treatment (n = 1; 6%), and continuing weight loss (n = 1; 6%).

### Intervention functions and behavior change techniques

**Fig. 1:**
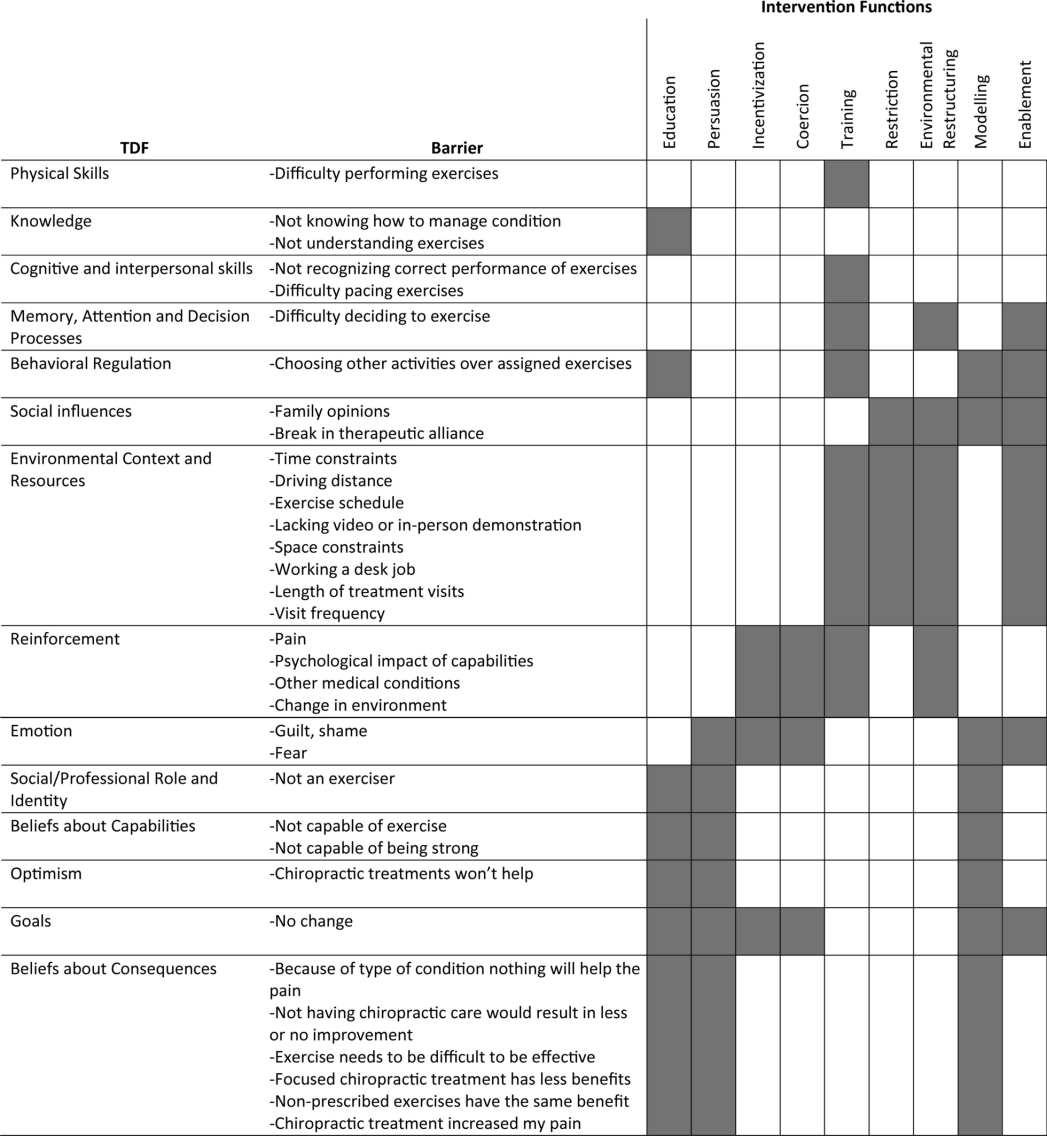
Intervention functions

Intervention functions found to be relevant to identified barriers are displayed in Fig. 1. All 9 IFs described in the BCW were identified. The most common IFs identified were modelling and education. Restriction, incentivization, and coercion were the least common intervention functions. No IFs linked to the TDF category ‘intentions’ were identified as no barriers existed in this domain. Behavior change techniques corresponding to each intervention function and considered appropriate by an author applying clinical judgment and the APEASE criteria are displayed in Table 5.

**Table 5:** Barriers mapped to behavior change techniques

Barrier (n = cases)	TDF component	Intervention function	Behavior change technique
Chiropractic treatments won’t help (9) No change/no goal (6) Not knowing how to manage condition (3) Because of type of condition nothing will help the pain (3) Not having chiropractic care would result in less or no improvement (3) Choosing other activities over assigned exercises (2) Not understanding exercises (1) Not an exerciser (1) Not capable of exercise (1) Not capable of being strong (1) Exercise needs to be difficult to be effective (1) Focused chiropractic treatment has less benefits (1) Non-prescribed exercises have the same benefit (1) Chiropractic treatment increased my pain (1)	Knowledge Behavioral regulation Social/professional role and identity Beliefs about capabilities Optimism Goals Beliefs about consequences	Education	Information about health consequences Feedback on behavior Feedback on outcome(s) of behavior Prompts/cues Self-monitoring of behavior Verbal persuasion to boost self-efficacy
Chiropractic treatments won’t help (9) No change/no goal (6) Because of type of condition nothing will help the pain (3) Not having chiropractic care would result in less or no improvement (3) Guilt, shame (2) Fear (1) Not an exerciser (1) Not capable of exercise (1) Not capable of being strong (1) Exercise needs to be difficult to be effective (1) Focused chiropractic treatment has less benefits (1) Non-prescribed exercises have the same benefit (1) Chiropractic treatment increased my pain (1)	Emotion Social/Professional role and identity Beliefs about capabilities Optimism Goals Beliefs about consequences	Persuasion	Information about health consequences Feedback on behavior Feedback on outcome(s) of the behavior Focus on past success Framing/reframing Verbal persuasion about capability
No change/no goal (6) Pain (5) Psychological impact of capabilities (2) Guilt, shame (2) Other medical conditions (1) Change in environment (1) Fear (1)	Reinforcement Emotion Goals	Incentivization	Feedback on behavior Feedback on outcome(s) of behavior Self-monitoring of behavior Discrepancy between current behavior and goal Remove adverse stimulus
No change/no goal (6) Pain (5) Psychological impact of capabilities (2) Guilt, shame (2) Other medical conditions (1) Change in environment (1) Fear (1)	Reinforcement Emotion Goals	Coercion	Feedback on behavior Feedback on outcome(s) of behavior Self-monitoring of behavior Discrepancy between current behavior and goal Incompatible beliefs
Time constraints (12) Pain (5) Difficulty performing exercises (4) Driving distance (3) Exercise schedule (3) Difficulty deciding to exercise (2) Choosing other activities over assigned exercises (2) Lacking video or in-person demonstration (2) Space constraints (2) Psychological impact of capabilities (2) Not recognizing correct performance of exercises (1) Difficulty pacing exercises (1) Working a desk job (1) Length of treatment visits (1) Visit frequency (1) Other medical conditions (1) Change in environment (1)	Physical skills Cognitive and interpersonal skills Memory, attention and decision processes Behavioral regulation Environmental context and resources Reinforcement	Training	Demonstration of the behavior Instruction on how to perform the behavior Feedback on the behavior Feedback on outcome(s) of the behavior Self-monitoring of the behavior Self-monitoring of outcome(s) of the behavior Behavior practice/rehearsal Habit formation Graded tasks
Time constraints (12) Driving distance (3) Exercise schedule (3) Lacking video or in-person demonstration (2) Space constraints (2) Family opinions (1) Break in therapeutic alliance (1) Working a desk job (1) Length of treatment visits (1) Visit frequency (1)	Social influences Environmental context and resources	Restriction	*No BCTs are linked through restriction
Time constraints (12) Pain (5) Driving distance (3) Exercise schedule (3) Difficulty deciding to exercise (2) demonstration (2) Space constraints (2) Psychological impact of capabilities (2) Family opinions (1) Break in therapeutic alliance (1) Lacking video or in-person Working a desk job (1) Length of treatment visits (1) Visit frequency (1) Other medical conditions (1) Change in environment (1)	Memory, attention and decision processes Social influences Environmental context and resources Reinforcement	Environmental restructuring	Adding objects to the environment Prompts/cues Restructuring the physical environment Restructuring the social environment
Chiropractic treatments won’t help (9) No change/no goal (6) Because of type of condition nothing will help the pain (3) Not having chiropractic care would result in less or no improvement (3) Choosing other activities over assigned exercises (2) Guilt, shame (2) Family opinions (1) Break in therapeutic alliance (1) Fear (1) Not an exerciser (1) Not capable of exercise (1) Not capable of being strong (1) Exercise needs to be difficult to be effective (1) Focused chiropractic treatment has less benefits (1) Non-prescribed exercises have the same benefit (1) Chiropractic treatment increased my pain (1)	Behavioral regulation Social influences Emotion Social/professional role and identity Belief about capabilities Optimism Goals Beliefs about consequences	Modeling	Demonstration of the behavior
Time constraints (12) No change/no goal (6) Driving distance (3) Exercise schedule (3) Difficulty deciding to exercise (2) Choosing other activities over assigned exercises (2) Lacking video or in-person demonstration (2) Space constraints (2) Guilt, shame (2) Family opinions (1) Break in therapeutic alliance (1) Working a desk job (1) Length of treatment visits (1) Visit frequency (1) Fear (1)	Memory, attention and decision processes Behavior regulation Social influences Environmental context and resources Emotion Goals	Enablement	Social support (unspecified) Social support (practical) Goal setting (behavior) Goal setting (outcome) Adding objects to the environment Problem solving Action planning Self-monitoring of behavior Restructuring the physical environment Restructuring the social environment Review behavior goal(s) Review outcome goal(s) Reduce negative emotions Verbal persuasion about capability

## Discussion

This study identified barriers and facilitators for participants engaging in BRLP self-management activities within the context of a controlled clinical trial, and linked barriers to theory-informed behavior change intervention elements. A previous qualitative study utilizing interviews from the same treatment arm of the larger controlled clinical trial explored participant satisfaction with and perceptions of the SMT + HEA treatment arm. When participants discussed their satisfaction, they considered study factors such as interactions with providers and research staff, perceived treatment effects of both the chiropractic treatments and home exercise program, structure of the exercise program, and information received [40]. Similar factors were identified in the current study including the positive social influence of providers, environmental atmosphere created by staff, beliefs that engaging in chiropractic treatment or exercise will reduce pain or result in general health progress and finding exercise handouts and the schedule as facilitating engaging in self-management. The current study utilizes a different lens allowing for mapping of identified factors using an established theory-informed method that moves findings towards practical application.

**Fig. 2:**
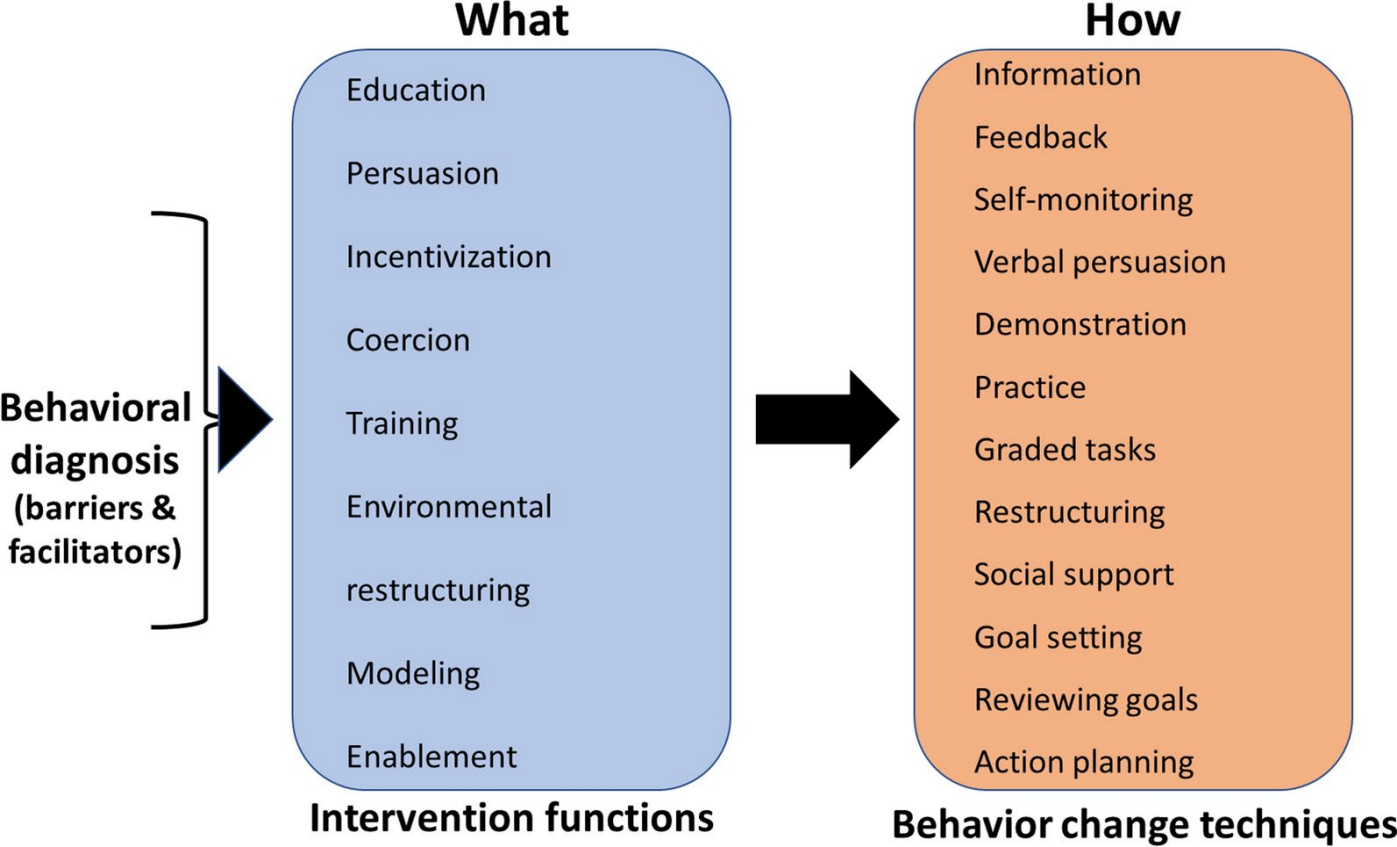
Common IFs and BCTs for addressing barriers to self-management for BRLP. Adapted from Michie et al.’s Behavioral Change Wheel [30]

This study utilized a theory-informed, established model for describing and intervention mapping addressing barriers and facilitators to self-managing BRLP. This process allowed establishing a behavioral diagnosis by identifying patient needs through barriers and facilitators, what is needed to meet their needs (IFs) and how this may be accomplished (BCTs) in a way facilitating behavioral change (Fig. 2) [30]. Our findings contribute implications for the role of providers engaging with persons experiencing BRLP.

Participants in our study were found to need education (“increasing knowledge or understanding”) and persuasion (“using communication to induce positive or negative feelings or stimulate action”) [30]. Linked BCTs reveal this can be accomplished through providing information such as the nature of chronic pain and BRLP, as well as the consequences of self-managing. Education and persuasion can also be affected through boosting patient’s self-efficacy and personal views of capability through verbal persuasion. Utilizing these techniques requires providers engage in clear communication as part of a strong provider-patient partnership [20, 23, 24, 47]. While education is commonly recommended by guidelines [15–18], our findings suggest greater emphasis is needed spanning more than general education about health conditions.

This study found participants also needed incentivization (“creating an expectation of reward”) and coercion (“creating an expectation of punishment or cost”) [30]. Linked BCTs demonstrate affecting incentivization and coercion may be achieved through providing feedback on behaviors and the outcomes of behavior [30]. This may include providing feedback on exercises, activities interfering with engaging with self-management, and behavior outcomes such as soreness or pain after exercising. While coercion is defined as “creating an expectation of punishment or cost” [30], in the current context it may be an avenue for providers being frank with patients regarding evidence-based information about long-term health costs of inactivity or unhealthy behaviors. Providers can also offer patients tools for facilitating self-monitoring of behaviors, including paper logs or web-based tools, or digital applications [48–50].

Participants were found to need training (“imparting skills”) [30]. The linked BCTs revealed accomplishing this, can occur through demonstrating the desired behavior, such as how to perform the exercise in addition to providing instructions. For instance, this study found participants believed the printed handouts were helpful, but some still required video or in-person demonstration. For example, providers should allow time and space for allowing patients to practice behaviors in office, facilitating immediate feedback on the behavior. Following up and monitoring in subsequent visits with additional feedback on both the behavior and outcome can also be used, requiring practitioners to check in with patients on a regular basis [51]. One technique is to employ graded tasks (e.g. choosing appropriate exercises and increasing doses, difficulty, etc.) that can also be used for gradually developing physical skills and overcoming fear of movement or pain [52]. Breaking exercises into achievable components is a person-centered approach, meeting patients where they are with the potential for reducing anxiety, increasing adherence, and trust in providers [53–56].

Enablement (“increasing means/reducing barriers to increase capability or opportunity”) was also needed [30]. Associated BCTs show affecting enablement could be done through social support, and problem solving. Additionally, providers can enable their patients by working with them to establish both behavioral and outcome driven goals. Once goals are created, reviewing goals and creating a plan of action can be utilized for facilitating the meeting of goals. One well-recognized method of goal setting involves the use of the acronym SMART; creating goals that are specific, measurable, achievable, relevant and time-bound [57, 58]. Enabling patients also occurs through the provision and facilitation of social support. Social support may be in the form of family, friends, or from the provider by establishing a strong therapeutic alliance with the patient through communication and responding to patient needs [59].

### Strengths

This study has several strengths including the use of a comprehensive and established behavioral model to aid in identifying BRLP patients’ behavioral needs and matching them to provider based solutions that could be applied in practice. It is also the first study exploring BRLP self-management from a behavioral perspective. Utilizing behavior change diagnoses in addition to the clinical diagnosis, permits development and testing of theory-informed self-management interventions with elevated methodological rigor and intervention fidelity. Utilizing the BCW approach consolidates the different definitions of self-management, used in this research [29, 60] and may improve the small effect sizes typically reported from self-management trials which precludes guideline authors from making strong recommendations on strategies to improve self-management by patients experiencing BRLP [19, 29].

### Limitations

The context and constraints of this study pose several limitations. This study was conducted as a secondary analysis. As such, the interview schedule was not designed for our specific research purpose. Different barriers and additional facilitators may have been identified with a tailored interview schedule. The depth and breadth of answers within the interviews were, at times, limited. This may have affected how often barriers and facilitators mapping to more complex TDF domains were identified. As a secondary analysis this study contained a singular data sources (interviews), and relies on documentation of the original study for describing the original research context and participants impacting the trustworthiness component. However, our sampling strategy of selected transcripts was random, methods were congruent with well documented theory informed processes, and coding involved peer debriefing and verification within consensus meetings.

Participants were provided 12 weeks of care within the context of a clinical trial, enrolling participants between the years 2007–10, which included informational resources, spinal manipulative therapy, and guided home exercises. While providers did individualize instruction within the constraints of the study protocol which included practice and spine positioning awareness related to their activities of daily living, these interventions may not reflect clinical care received in community-based settings or within today’s current sociocultural context.

## Conclusion

This study identified barriers and facilitators to engaging in self-management for participants in a pragmatic, randomized clinical trial. A rigorous systematic intervention mapping process utilizing a theory-informed behavior change approach, Michie et al.’s BCW was used for describing what participants need and how their needs may be met [21]; providing insight into the role back pain providers had in facilitating self-management. These findings may support the design of future self-management interventions for persons experiencing BRLP.

## Supplementary Information

Additional file 1

Additional file 2

## Data Availability

No datasets were generated or analysed during the current study.

## Abbreviations

LBP: Low back pain
BRLP: Back-related leg pain
CPG: Clinical practice guideline
BCW: Behavior change wheel
SMT: Spinal manipulative therapy
HEA: Home exercise and advice
SRQR: Standards for Reporting Qualitative Research
TDF: Theoretical Domains Framework
COM-B: Capability, Opportunity, Motivation Behaviour Model
IF: Intervention function
APEASE: Affordability, practicability, effectiveness and cost-effectiveness, acceptability, side effects/safety and equity
BMI: Body mass index
SMART: Specific, measurable, achievable, relevant and time-bound
