# Supplementary material for: Barriers and facilitators to self-management in people with back-related leg pain: a qualitative secondary analysis
Source: Chiropr Man Therap. 2025 May 5;33:17. doi: 10.1186/s12998-025-00578-z (PMC12054131; doi:10.1186/s12998-025-00578-z)
Supplement: Supplementary file 1 — Additional file 1 [file 12998_2025_578_MOESM1_ESM.docx]

| Interview Schedule | | | |
| --- | --- | --- | --- |
| Question 1. | | When we asked you, “overall, how satisfied are you with the care you received in the study,” what things did you take into account when deciding how satisfied you were? | |
| Question 2. | | When we asked you, “overall, how much your leg or low back pain changed,” what things did you consider when answering that question? | |
| Question 3. | | Now that you are finished with treatment in the study, is your level of improvement what you expected? | |
| Question 4. | | What did you like the best about the: | |
|  | Question 4a. |  | home exercise program you had in the study? |
|  | N/A or Question 4b. |  | chiropractic treatments you had in the study? |
| Question 5. | | What did you like the least about the: | |
|  | Question 5a. |  | home exercise program you had in the study? |
|  | N/A or Question 5b. |  | chiropractic treatments you had in the study? |
| Question 6. | | Overall, did you feel like the care you received in the study was worthwhile? | |
|  | (If YES): |  | Why was it worthwhile? |
|  | (If NO): |  | Why wasn't it worthwhile? |
