# Supplementary material for: Barriers and facilitators to self-management in people with back-related leg pain: a qualitative secondary analysis
Source: Chiropr Man Therap. 2025 May 5;33:17. doi: 10.1186/s12998-025-00578-z (PMC12054131; doi:10.1186/s12998-025-00578-z)
Supplement: Supplementary file 2 — Additional file 2 [file 12998_2025_578_MOESM2_ESM.docx]

Appendix 2: Codebook

|  |  | **Themes (Nodes)** | **Operational Definitions** | **Example words/phrases** |
| --- | --- | --- | --- | --- |
| **COM-B** | | **TDF** |  |  |
| Capability | Physical | Physical Skills | individual mentions ability and proficiency for a physical skill acquired through practice impacting self-managing (symptoms, treatments, etc.) [an ability of proficiency acquired through practice] Example - I am able to perform glute bridges | Barrier: mentions inability to do for exercises, stretches, mobility, postural adjustment Facilitator: mentions ability to do exercises, stretches, mobility, postural adjustment |
|  | Psychological | Knowledge | individual mentions an awareness of the existence of something impacting self-managing (symptoms, treatments, etc.) [an awareness of the existence of something] Example - knowing instructions on how to lift and bend | knowing how to do exercises, stretches, postural adjustment; factual knowledge, procedures, rationale; "I should do this" |
|  |  | Cognitive and interpersonal skills | individual mentions an ability or proficiency in a cognitive skill acquired through practice impacting self-managing (symptoms, treatments, etc.) [an ability of proficiency acquired through practice] Example - I am getting better at recognizing when I need a modification to an exercise and communicating that to the instructor. 2nd example - I am more aware of pacing myself when exercising. | awareness or self-assessment of how you're doing; recognizing and communicating a need; "my provider listened to my concerns and modified treatment" |
|  |  | Memory, Attention and Decision Processes | Individual mentions retaining information, gaining or maintaining focus, or making decisions pertaining to self-managing (symptoms, treatments, etc.) [the ability to retain information, focus selectively on aspects of the environment and choose between 2 or more alternatives] Example: remembering instructions on moving when doing activities; using ice or heat when noticing the beginning of a pain flare | deciding to do exercises or self-management activities; remembering to be aware; noticing and taking the next step |
|  |  | Behavioral Regulation | Individual mentions something pertaining to managing or changing self-managing behaviors [anything aimed at managing or changing objectively observed or measured actions] Example - when I had the urge to take a pain medication, I exercised instead | awareness of maladaptive behavior or thoughts leading to changing behavior towards self-management; self-monitoring, breaking habit |
| Opportunity | Social | Social influences | Individual mentions interpersonal interaction or influence causing their thoughts, feelings, or behaviors towards self-managing (symptoms, treatment, etc.) to change. [those interpersonal processes that can cause individuals to change their thoughts, feelings, or behaviors] Example: Mentions family member encouraging them to seek care, so they made an appointment. Negative example - spouse doens't believe back pain is severe or needs treatment | therapeutic alliance; because of 'any social INFLUENCE' then…thoughts, behavior, feelings (interaction with other person or persons--updated 6_17_2022) |
|  | Physical | Environmental context and resources | Individual mentions something in their personal situation or environment discouraging or encouraging the development of skills, abilities, independence, social competence, and adaptive behavior for self-managing (symptoms, treatments, etc.) [any circumstance of a person's situation or environment that discourages or encourages the development of skills and abilities, independence, social competence, and adaptive behavior] Example, negative - the clinic was too far awary so I didn't going in for treatments; | having space to exercise, environment of clinic, infrastructure, material resources (including exercises--i.e., graded exercises--updated 6_17_2022), organisational culture (of the clinic--updated 6_17_2022); pertains to both treatments and at-home managing |
| Motivation | Automatic | Reinforcement | Individual mentions something increasing the probability of self-managing behaviors (symptoms, treatment, etc.) via a dependent relationship, or contingency, or responding to a stimulus. [increasing the probability of a response by arranging a dependent relationship, or contingency, between the response and a given stimulus] Example- Effectiveness of treatment encouraging continuing of treatment | cause and effect relationship; stimulus=outcome or something done encouraging behavior; self-management or treated related; exercises felt good |
|  |  | Emotion | Individual mentions a reaction pattern involving a experiential, behavioral, or physiological element stemming from or impacting self-managing (symptoms, treatment, etc.) [a complex reaction pattern, involving experiential, behavioral, and physiological elements, by which the individual attempts to deal with a personally significant matter or event] Example, negative - Individual's expresses frustration due to viewing exercises as repetitive and rigorous causes them to change exercise schedule | emotional words expressed - I feel frustrated, bored, silly, stressed, hopeful |
|  | Reflective | Social/Professional Role and Identity | Individual mentions personal behaviors and/or qualities within a social or work setting impacting self-managing (symptoms, treatment, etc.) [a coherent set of behaviors and displayed personal qualities of an individual in a social or work setting] Example, negative - Because I am a women, I don't feel comfortable exercising at the gym because I don't like being watched and its all men working out. Example - all my friends lift weights, so I feel out of place just doing yoga stretches. | usually organizational level; mentions connection to social role or identity "I am this"; belonging to a group and receiving either acceptance or marginalization |
|  |  | Beliefs about Capabilities | Individual mentions accepting truth, reality, or validity about their ability, talent, or facility for self-managing (symptoms, treatment, etc.) [acceptance of the truth, reality, or validity about an ability, talent, or facility that a person can put to constructive use] Example - I am now confident I can walk a mile. | confidence in abilities, lack of fear, I'm not able, I can't; leading to ability or belief to do something; Retrospective or present or prospective: have been able to do AND/OR belief that an action in the future is possible |
|  |  | Optimism | Individual mentions confidence in outcome, attaining goals, or physical or mental capabilities for self-managing (symptoms, treatment, etc.) [the confidence that things will happen for the best or that desired goals will be attained] Example, negative - I am not confident the treatment will help me get out of pain. | confidence tied to an end result or goal - health, pain |
|  |  | Intentions | Individual mentions a conscious decision for performing a behavior, resolving to think or act in a certain way related to self-managing (symptoms, treatment, etc.) [a conscious decision to perform a behavior or a resolve to act in a certain way] Example- I have decided to exercise 3 times a week to help relieve my pain. | I have decided to do, I will do, will not do |
|  |  | Goals | individual mentions a mental representation of outcomes or end states they want to achieve for self-managing (symptoms, treatment, etc.) [mental representations of outcomes or end states that an individual wants to achieve] Example- I want my leg pain to improve. | an end goal, result, outcome is mentioned wanting to be worked towards |
|  |  | Beliefs about consequences | Individual mentions accepting truth, reality, or validity about outcomes of a physical or cognitive behavior in pertaining to self-managing (symptoms, treatment, etc.) [acceptance of the truth, reality, or validity about outcomes of a behavior in a given situation] Example- if I exercise the pain won't go away completely, but there will be improvement in performing daily activities | If I do this, why do I enact behavior; has outcome |
